# IgY Antibodies Protect against Human Rotavirus Induced Diarrhea in the Neonatal Gnotobiotic Piglet Disease Model

**DOI:** 10.1371/journal.pone.0042788

**Published:** 2012-08-03

**Authors:** Celina G. Vega, Marina Bok, Anastasia N. Vlasova, Kuldeep S. Chattha, Fernando M. Fernández, Andrés Wigdorovitz, Viviana G. Parreño, Linda J. Saif

**Affiliations:** 1 Instituto de Virología, CICV y A - INTA Castelar, Buenos Aires, Argentina; 2 Food Animal Health Research Program (FAHRP), Ohio Agricultural Research and Development Center, Veterinary Preventive Medicine Department, The Ohio State University, Wooster, Ohio, United States of America; INRA, UR1282, France

## Abstract

Group A Rotaviruses are the most common cause of severe, dehydrating diarrhea in children worldwide. The aim of the present work was to evaluate protection against rotavirus (RV) diarrhea conferred by the prophylactic administration of specific IgY antibodies (Ab) to gnotobiotic piglets experimentally inoculated with virulent Wa G1P[8] human rotavirus (HRV). Chicken egg yolk IgY Ab generated from Wa HRV hyperimmunized hens specifically recognized (ELISA) and neutralized Wa HRV *in vitro*. Supplementation of the RV Ab free cow milk diet with Wa HRV-specific egg yolk IgY Ab at a final ELISA Ab titer of 4096 (virus neutralization –VN- titer = 256) for 9 days conferred full protection against Wa HRV associated diarrhea and significantly reduced virus shedding. This protection was dose-dependent. The oral administration of semi-purified passive IgY Abs from chickens did not affect the isotype profile of the pig Ab secreting cell (ASC) responses to Wa HRV infection, but it was associated with significantly fewer numbers of HRV–specific IgA ASC in the duodenum. We further analyzed the pigś immune responses to the passive IgY treatment. The oral administration of IgY Abs induced IgG Ab responses to chicken IgY in serum and local IgA and IgG Ab responses to IgY in the intestinal contents of neonatal piglets in a dose dependent manner. To our knowledge, this is the first study to show that IgY Abs administered orally as a milk supplement passively protect neonatal pigs against an enteric viral pathogen (HRV). Piglets are an animal model with a gastrointestinal physiology and an immune system that closely mimic human infants. This strategy can be scaled-up to inexpensively produce large amounts of polyclonal IgY Abs from egg yolks to be applied as a preventive and therapeutic passive Ab treatment to control RV diarrhea.

## Introduction

Group A rotaviruses (RVA) are the most common cause of severe, dehydrating diarrhea in children worldwide. Diarrhea causes 1.3 million deaths in children younger than 5 years annually [1], [2]. Rotavirus surveillance studies indicate that all young children experience at least one RVA infection by 5 years of age [3], [4]. Moreover, RV infection is responsible for about 30% of all hospital admissions for diarrheal disease and causes an estimated of 400,000–600,000 deaths per year among children worldwide [5]. Most of these deaths occur in developing countries in Asia and sub-Saharan Africa, where human (H) RV is the leading cause of life-threatening diarrhea in infants and young children [3], [4]. The National Rotavirus Surveillance Program reported almost 120,000 cases of diarrhea and 150 deaths associated with RV in children under 5 years old in Argentina [6].

The five most prevalent G (VP7) and P (VP4) genotypes worldwide include G1, G3, G4, and G9 with P[8] and G2 P[4] [7]. The virus infects the mature villus epithelial cells of the small intestine and infection often leads to fever, vomiting and diarrhea in children. Dehydration and electrolyte disturbances are the major sequelae of HRV infection and occur most often in the youngest children [8], [9]. The high morbidity and mortality related with HRV gastroenteritis makes its prevention and treatment an important public-health goal. A specific, effective and affordable therapy is currently not available [10]. However, two RVA vaccines were licensed in 2006. Rotarix® is a monovalent live attenuated vaccine derived from the most common circulating wild-type strain G1P[8] (Glaxo SmithKline Biologicals, Belgium) and RotaTeq® is composed of 5 RV strains, each of which is a single-gene reassortant based on a parent bovine strain -WC3- that contains an outer capsid gene from a human strain that induces immunity to the most common genotypes of HRV in circulation −G1 to G4− and P1A [8] (Merck & Co., USA). Both vaccines were efficacious in preventing severe gastroenteritis, predominantly due to the G1 HRV serotype [8], [11], [12], in developed countries. However in children from developing countries, the efficacy rates were lower [13]. There is also evidence of vaccine-acquired RVA infection in children with severe combined immunodeficiency [14]. Studies of candidate vaccines, passive antibody (Ab) treatments and development of immune responses to HRV can be conducted in the neonatal gnotobiotic (Gn) pig model, the only animal model susceptible to HRV infection and disease [15], [16]. Furthermore, the gastrointestinal physiology and immune system of the neonatal Gn piglets resemble those of human infants, so responses detected in neonatal pigs closely mimic those observed after natural HRV infection of infants in terms of the pathogenicity of HRV challenge [15], [17]. The animals are colostrum-deprived, maintained in isolator units and fed sterile milk, assuring a pathogen/microbe-free and Ab-free status, making them an ideal model to study active or passive immunity *in vivo* [15], [16], [18], [19], [20], [21].

Regarding the immune correlates of protection from RVA infection and disease it is now known that both humoral and cell-mediated immunity are important in the resolution of ongoing RVA infection and in protection against subsequent infection [9]. Several studies of immune responses to RV infection and vaccination and associated correlates of protection have been carried out in human and experimental animal models and have been reviewed elsewhere [15], [17], [20], [22]. The primary infection elicits a predominantly homotypic, serum-neutralizing Ab response to the virus and subsequent infections elicit a broader, heterotypic response. Local immunity in the gut, specially the presence of IgA Abs, proved to be highly important for protection in humans [8]. Furthermore, protection against RV diarrhea in children seems to be serotype specific and related to the levels of neutralizing Ab against the homotypic virus where local IgA Abs may play a critical role [22]. In the gnotobiotic pig model of HRV infection, previous studies demonstrated that pigs recovered from virulent HRV infection are completely protected from virus shedding upon challenge. The protection rates from this group of pigs were positively correlated with the magnitude of IgA Ab secreting cell and B-cell responses in the intestinal lamina propria [15], [20]. Mice are other experimental animals in which RV infections have been studied. Mouse pups can be infected with murine RV and develop disease while adult mice are refractory to RV diarrhea and are only a RV shedding model [17]. In adult mice, the systemic or mucosal production of IgG and IgA Abs are critical for clearing RV infection [17]. Studies of mice with different immunological knockout lesions have demonstrated that multiple humoral and innate immune mechanisms contribute to protection from RV infection [22], [23].

The oral administration of virus-neutralizing Abs is an attractive approach for passive protection of humans and animals against gastrointestinal pathogens [10], [18], [24], [25], [26]. Studies using immune bovine colostrum demonstrated that this preventive passive treatment significantly reduced the risk for RVA gastroenteritis during an outbreak in children and the number of days with RVA-associated diarrhea [27], [28]. On the other hand, IgY egg yolk immunoglobulins derived from hyperimmunized hens represent an economically feasible and practical strategy which has been explored for the passive treatment of RVA infection and diarrhea [29]. The IgY technology offers several advantages over other methods of Ab production [30]. This is a non-invasive technology and IgY Abs do not activate mammalian complement and do not bind protein A or G [31]. Furthermore, IgY Abs are the only Ab isotype present in chicken egg yolk, so its extraction is simple, and approximately 1500 mg of IgY can be harvested each month from each laying hen (5–25 mg/egg yolk), with between 2 to 10% being antigen-specific IgY [30]. These properties make IgY production a faster and cheaper method for polyclonal Ab production than from other sources [32], [33], [34].

Furthermore, eggs contain higher levels of Abs per ounce than milk does [35]. In addition, it has been demonstrated in pre-clinical studies that eggs of hyperimmunized hens possessed anti-inflammatory and anti-diarrheic properties and offer benefits to a number of different human structures and functions including the circulatory and immune systems and the joints [35]. Akita and co-workers showed that egg yolk as well as purified IgY Abs were immunogenic but failed to induce a detectable IgE Ab response in intraperitoneally immunized mice [36]. In this study we evaluated the protection against HRV diarrhea conferred by the prophylactic administration of HRV specific IgY Abs to gnotobiotic piglets experimentally inoculated with the prevalent strain of HRV, Wa G1P[8].

## Materials and Methods

### Ethics statement

This study was approved by the Institutional Animal Care and Use Committee (IACUC) of the Ohio State University, and conducted in compliance with local and federal guidelines. Animals were carefully monitored for any signs of pain or distress and the procedures to ameliorate suffering are described in the protocol in detail. Oral inoculation of young pigs with VirHRV caused only transient clinical disease with diarrhea of variable severity from which piglets recovered spontaneously within a few days. The euthanasia was performed by electrocution following anesthesia.

### Preparation of antibody pools

#### Wa HRV and VP6 IgY Abs from egg yolk

The egg yolk pools used in this study were obtained from immunized and non-immunized hens. Eight-week-old birds were bought and housed in cages specially designed for this purpose in groups of two animals per cage following animal welfare recommendations [37], [38]. Room temperature, relative humidity and light/dark cycles were controlled. Hens were fed laying hen diet and provided water *ad libitum*, and eggs were collected daily starting at the first immunization. To generate the specific IgY Abs from egg yolk, 20 Lohmann Brown Classic laying hens were hyperimmunized with Wa HRV and 10 hens were hyperimmunized with recombinant viral protein 6 (VP6) from RV. This viral protein was obtained by recombinant baculovirus (containing the VP6 gene from C486 strain of bovine RV (SbI P[1]G6); kindly supplied by L. Babiuk, VIDO, Canada) infection of Sf9 cells. The attenuated Wa HRV (P[8]G1) with the titer of 10^7^ focus forming units (FFU)/ml or recombinant VP6 (ELISA titer to Wa HRV: 10^−4^; approximately 150 µg/injection) were mixed in a ratio of 1∶1 in Freund´s complete adjuvant (FCA) or Freund´s incomplete adjuvant (FIA; both from Sigma-Aldrich, Germany). Birds were immunized intramuscularly in the breast tissue with 500 µl of immunogen. The FCA was used only for the first immunization, while FIA was used for the subsequent booster injections. Based on previous studies carried out in our laboratory, we followed the optimal immunization regimen where animals received the first immunization before starting egg production. There were no side effects on the onset of laying. Serum samples and egg yolks were collected and IgY Ab titer to Wa HRV was determined by ELISA. Pools of egg yolks from the immunized hens were prepared weekly and were diluted in distilled water at a ratio of 1∶3 and freeze-thawed to separate the emulsion. Antibodies were precipitated following an ammonium sulphate precipitation protocol. Briefly, water diluted egg yolks were centrifugated at 8,000 g for 12 min at 4°C to remove lipids. The supernatant was then incubated with 0.24 g/ml of ammonium sulphate (Anedra S.A., Argentina) for 10 min at room temperature and then centrifugated at 10,000 g for 12 min at 4°C. The pellet was resuspended in 2 M ammonium sulphate solution at the original egg yolk volume and incubated at room temperature for 10 min. The solution was then centrifuged at 10,000 g for 12 min at 4°C and the pellet was resuspended in phosphate saline buffer pH 7.4 (PBS) at a 1∶10 ratio of the original volume of the egg yolk pool. This solution was dialyzed against PBS, sterilized by filtration (0.22-µm-pore-size membrane filter; Millipore, USA), and stored at −20°C until used. IgY Ab titer was determined by ELISA and virus neutralization (VN) assays. The purity of these preparations was evaluated by SDS-PAGE followed by Coomasie blue staining and Western blot. Protein concentration was measured by spectrophotometry at 280 nm (NanoDrop 1000, Thermo Scientific, USA) and total IgY concentration was determined by ELISA.

Control IgY pool was prepared following the same protocol and was also resuspended in phosphate saline buffer pH 7.4 (PBS) at a 1∶10 ratio of the original volume of the egg yolk pool. Although all the pellets were resuspended in a 1∶10 ratio of the original volume of egg yolk, they were then dialyzed against PBS pH 7.4, so the final volumes recovered were different from the initial ones.

Purified Ab batches from Wa HRV hyperimmunized hens with IgY Wa HRV Ab titers of 16,384 by ELISA were mixed and designated as Wa HRV IgY POOL A while batches with IgY Ab titers of 65,536 were mixed to generate the Wa HRV IgY POOL B. Purified Ab batches from VP6 hyperimmunized hens with IgY Wa HRV Ab titers of 65,536 were designated VP6 IgY POOL. Finally, egg yolks from non-immunized hens processed in a similar manner were designated CONTROL IgY POOL which was used as negative control.

#### Wa HRV IgG Abs from immune sow serum

RVA-seropositive sows (n = 2) received five doses of attenuated Wa HRV (∼107 FFU/dose) intramuscularly at 2-week intervals, with the first dose in FCA, followed by three doses in FIA and the last one without any adjuvant. A week after the last immunization, serum was collected and pooled. Antibodies were precipitated using the same ammonium sulphate protocol as previously described for IgY Abs and then filtered (0.22-µm-pore-size membrane filter; Millipore, USA), and stored at −20°C until used. Titers of VN and ELISA Ab titers to Wa HRV in this pool were determined. Purified IgG from sow serum served as the positive source of homologous Abs to Wa HRV (Wa HRV IgG pool).

### Virus

Virulent (Vir) Wa HRV (G1, P1A [8] containing intestinal contents of gnotobiotic pigs were diluted in minimal essential medium (MEM; Invitrogen, USA), and used for virus inoculation as described elsewhere [18]. Attenuated Wa HRV (cell culture adapted) was propagated in monkey kidney (MA- 104) cells for use in vaccine formulation, enzyme-linked immunospot assay (ELISPOT), ELISA and VN assays.

### Continuous cell lines

The MA-104 cell line of fetal rhesus monkey kidney cells (passage 36) was originally bought from Microbiological Associates (now called BioWhittaker) and maintained in our lab since 2004. The Sf9 cell line, a clonal isolate derived from *Spodoptera frugiperda* (Fall Armyworm), was kindly provided by Dr. K.O. Chang and maintained in our lab since 2002.

### Gnotobiotic pigs: Experimental design, virus inoculation, clinical observations and sample collection

#### Gnotobiotic pigs

Gnotobiotic pigs were derived by hysterectomy of near-term sows and maintained in isolator units as previously described [21], [39]. Pigs were allocated to one of six groups (Gp1 to 6) as detailed in Table 1. ELISA Ab titer to Wa HRV was used as the adjusting parameter to compare the non-neutralizing VP6 IgY Abs as a treatment. However, Wa HRV IgY 4096 and Wa HRV IgG 4096 had the same VN titer in milk (VN: 256). During the first 24 h of life, piglets received commercial sterilized bovine milk (RV Ab free) for human consumption (Parmalat, USA) *ad libitum*. At 24 h of age, pigs in Gp1 to 4 received supplements of the corresponding IgY HRV Abs while pigs in Gp5 received porcine IgG HRV Ab, twice a day from 3 to 12 days of age (9 days of passive Ab treatment). Group 6 pigs received no Ab treatment and were fed only the commercial milk. The milk diet consisted on 210 ml of RVA Ab free commercial milk twice a day supplemented with the corresponding volume of HRV Ab pool, as described in Tables 1 and 2, representing approximately 4.8% of the milk diet (Table 1). Animals assigned to passive treatment groups started the treatment after the first 24 h of life. This was in the last 12 h prior to gut closure at an estimated 36 h of age, in order to reduce systemic Ab absorption [40], [41]. From day 13 of life onwards, after the end of the passive treatments, all pigs were fed only the Ab free milk twice a day until the end of the experiment at 21 post inoculation days (PID).

**Table 1:** Virus neutralization (VN) and ELISA isotype-specific Ab titers to Wa HRV in the supplemented milks used to feed gnotobiotic piglets.

Treatment group	Volume of the corresponding pool added to 210 ml of sterile Ab free milk*	Ab titer to HRV Wa P[8]G1	
		VN#	ELISA
**Gp1: Wa HRV IgY 1024 treatment**	10 ml of Wa HRV IgY pool A	**1024**	64
**Gp2: Wa HRV IgY 4096 treatment**	10 ml of Wa HRV IgY pool B	**4096**	256
**Gp3: VP6 IgY 4096 treatment**	10 ml of VP IgY pool	**4096**	64
**Gp4: CONTROL IgY treatment**	10 ml of control IgY pool	**<4**	<4
**Gp5: Wa HRV IgG 4096 treatment**	10 ml of Wa HRV IgG pool	**4096**	256
**Gp6: no Ab treatment**	-	**<4**	<4

# determined by fluorescence focus forming unit reduction assay.

* Volume of Ab' pools (control/Wa HRV/VP6) added to 210 ml of sterile commercial milk to obtain the indicated final Ab titer to Wa HRV. These volumes represented 4.8% of the total milk volume (210 ml). Ab titers of <4 are considered negative.

**Table 2:** Pools of Wa HRV specific porcine IgG Abs, VP6 specific and Wa HRV specific chicken egg yolk IgY Abs and control IgY Abs used for this experiment.

Pool	N° of egg yolks	Volume (lt)	Protein concentration (mg/ml)	Total IgY concentration (mg/ml)	ELISA titer	VN titer
**Control IgY pool**	*349*	1.36	10.4	9.4	256	64
**Wa HRV IgY pool A**	*218*	0.85	5.5	2.6	16,384	4096
**Wa HRV IgY pool B**	*513*	2.00	25.8	14.3	65,536	16,384
**VP6 IgY pool**	*220*	0.85	37.5	16.0	65,536	64
**Wa HRV IgG pool**	-	1.60	52.4	-	65,536	16,384

Twenty Lohmann Brown Classic laying hens were hyperimmunized with 10^7^ UFF/ml of Wa HRV (G1, P1A [8]) each time and 10 hens were hyperimmunized with recombinant VP6, 7 times. Crude egg yolks were diluted in distillated water in a 1∶3 ratio and precipitated using ammonium sulfate. Sow serum was also salt-precipitated. The pellets were suspended in 10% of the original volume of egg yolk or sow serum in PBS. The protein concentration was determined by spectrophotometry (Abs 280 nm; NanoDrop ND-1000, USA). The chicken IgY and porcine IgG Ab titers to Wa HRV G1P[8] were determined by ELISA and VN assays before and after sterilization by filtration (0.22-mm-pore-size membrane filter; Millipore, USA). The total IgY concentration was determined by ELISA.

#### Pig virus inoculation, clinical observations and sample collection

At 72 hours of age, pigs in all groups received 5 ml of 100 mM sodium bicarbonate (to neutralize gastric acidity), followed by 10^6.7^ FFU of virulent HRV Wa (VirWa) HRV in 5 ml of MEM (Invitrogen, USA) [18]. After VirWa HRV inoculation, piglets were examined daily for diarrhea and virus shedding from 0 to 21 PID. To estimate the severity of the diarrhea, fecal consistency was scored by qualified technicians as follows: 0: normal; 1: pasty; 2: semi-liquid; 3: liquid. A score equal or greater than 2 was considered diarrhea. Prior and after VirWa HRV inoculation, rectal swabs were collected daily to assess virus shedding. Briefly, swabs were resuspended in 8 ml MEM (Invitrogen, USA), corresponding to a 1∶25 dilution. All the samples were tested by ELISA for assessment of antigen shedding and by cell culture immunofluorescence assay for detection of infectious virus shedding. Serum samples were collected before the beginning of the passive treatments (within 24 h after birth), at virus inoculation, and then weekly (7, 14 and 21 PID). Serum Abs to Wa HRV were measured by isotype-specific ELISA and VN assays. The presence of coproantibodies (coproAb) was also assessed by ELISA. At 21±3 PID, the animals were euthanized to study the primary Ab responses to Wa HRV and HRV specific Ab secreting cells (ASC) were quantified by ELISPOT assay in the following gut-associated lymphoid tissues (GALT): duodenum, jejunum and ileum lamina propria (LP) and mesenteric lymph nodes (MLN); and in systemic lymphoid tissues: spleen and blood (PBL). Large (LIC) and small (SIC) intestinal contents from all the piglets were collected at necropsy for coproAb detection by ELISA [18], [19].

### Rotavirus antigen and viral detection

Wa HRV shedding was detected in rectal swabs using an antigen capture ELISA as described previously [18], [19], [42]. Virus infectious titer was assessed by a cell culture immunofluorescence (CCIF) assay as described previously [43]. Fluorescent cells were counted using a fluorescence microscope and titers were expressed as the number of fluorescent focus forming units per ml (FFU/ml).

### Porcine isotype-specific Ab to Wa HRV ELISA

The IgM, IgA and IgG Ab titers to Wa HRV were quantified in the Wa HRV porcine IgG Ab pool, pig sera, rectal swabs, large and small intestinal contents (LIC and SIC, respectively). Specific Abs to HRV were detected by an indirect ELISA using the reagents and protocol described previously [18], [19], [21].

### Wa HRV IgY specific Ab ELISA

The IgY Ab titers to Wa HRV were determined in hen sera, crude egg yolks, purified IgY pools, supplemented milks and pig sera, rectal swabs, LIC and SIC by an indirect ELISA. Briefly, 96 well ELISA plates (Maxisorp, NUNC, Denmark) were coated with hyperimmune antiserum to RV prepared in guinea pigs and then incubated with 10% chicken egg white albumin in PBS-Tween_20_ 0,05% (Sigma-Aldrich, Germany) for blocking. The supernatants of Wa HRV-infected (10^5^ FFU/ml) MA104 cell culture lysates or mock-infected MA104 cell lysates were then added, followed by serial four-fold dilutions of the samples. The reaction was developed using a peroxidase labeled polyclonal goat anti-chicken IgY Ab at a 1∶5000 dilution in PBS-Tween 0,05% (Sigma-Aldrich, Germany), as conjugated Ab and hydrogen peroxide and ABTS as susbtrate/cromogen system (KPL, Kirkegaard & Perry Laboratories Inc., USA).

### Total IgY ELISA

The total IgY concentration was determined in all the purified IgY pools used for this experiment. Briefly, 96 well ELISA plates (Polysorp, NUNC, Denmark) were coated with polyclonal goat anti chicken IgY (Sigma-Aldrich, Germany) at a 1∶5000 dilution in carbonate-bicarbonate coating buffer pH 9.6 and then incubated with 10% nonfat milk in PBS-Tween 0,05% for blocking. Serial five-fold dilutions of each purified IgY Ab pool (Wa HRV IgY pool A and B, VP6 IgY pool and control IgY pool) were incubated for 1 h at 37°C. As a standard of known concentration, affinity purified IgY from serum was included (Sigma-Aldrich, Germany) The plates were later incubated with commercial HRP-labeled goat polyclonal Ab to chicken IgY at 1∶1500 dilution (Sigma-Aldrich, Germany) for 1 h at 37°C. Hydrogen peroxide and ABTS were used as susbtrate/cromogen system (KPL, Kirkegaard & Perry Laboratories Inc., USA).

### Porcine IgG and IgA Ab ELISA to chicken IgY

The IgG Ab titers to chicken IgY were quantified in the pig sera, LIC and SIC. The IgA Ab titers to IgY Ab were quantified only in LIC and SIC. Briefly, 96 well ELISA plates (Maxisorp, NUNC, Denmark) were coated with 1 µg per well of commercial purified IgY (JacksonImmunoResearch Laboratories Inc., USA) and then incubated with 10% nonfat milk in PBS-Tween 0,05% for blocking. Serial four-fold dilutions of porcine serum or intestinal content samples were incubated for 1 h at 37°C. The plates were later incubated with commercial HRP-labeled goat polyclonal Abs to porcine IgA at 1∶3000 dilution (AbD Serotec Inc., USA) or with commercial biotin-labeled goat polyclonal Abs to porcine IgG at 1∶20,000 dilution (KPL, Kirkegaard & Perry Laboratories Inc., USA) for 1 h at 37°C. The wells incubated with commercial anti-pig IgG Ab were later incubated with commercial HRP-labeled streptavidin (1∶10,000) (Sigma-Aldrich, Germany) for 1 h at 37°C. The reaction was developed using a commercial HRP-labeled streptavidin (1∶10,000) (Sigma-Aldrich, Germany) for 1 h at 37°C. Hydrogen peroxide and ABTS were used as susbtrate/cromogen system (KPL, Kirkegaard & Perry Laboratories Inc., USA).

### Fluorescent focus reduction virus neutralization (FFN) test

VN Ab titers to Wa HRV in purified pools of Abs, egg yolks, supplemented milks and pig sera were determined by fluorescent focus neutralization (FFN) test as previously described [19]. The VN titer was expressed as the reciprocal of the highest sample dilution that resulted in >80% reduction in the number of fluorescent foci.

### Isolation of mononuclear cells (MNC) and Wa HRV-specific ELISPOT assay

Approximately 10–18 inches of tissue samples of duodenum, jejunum, and ileum lamina propria were collected. The mesenteric lymph nodes (MLN) were collected and processed separately. Mononuclear cells from blood and spleen were extracted to evaluate ASC responses in systemic lymphoid tissues. All the MNC suspensions were obtained as previously described for pig tissues [21] and the purified cells from all tissues were resuspended to a final concentration of 5×10^6^ MNC/ml in RPMI-1640 (GIBCO, USA) supplemented with 10% FCS, 20 mM HEPES, 2 mM Glutamine, 1 mM sodium pyruvate, 0.1 mM non-essential aminoacids, 100 IU/ml penicillin, 67 mg/ml streptomycin and 50 mg/ml gentamycin (E-RPMI). The cell viability of each MNC suspension was assessed by Trypan blue exclusion (in all cases it was >90%). An ELISPOT assay for quantification of Wa HRV specific IgM, IgA, IgG ASC was conducted to evaluate effector B-cell responses from all piglets at 21 PID, as previously described [18], [19], [21]. The ELISPOT plates were incubated for 2 h at 37°C with the following set of Abs: commercial HRP-labeled goat polyclonal Abs to porcine IgA (1∶1000) from AbD Serotec Inc., USA; commercial HRP-labeled goat polyclonal Abs to porcine IgM (1∶2000) (from AbD Serotec, USA) and biotinylated Mab from ascites fluid to pig IgG (hybridoma 3H7) (1∶10,000; 0.03 mg/ml) [19]. The wells incubated with anti-pig IgG mAb were later incubated with commercial HRP-labeled streptavidin (1∶10,000) (Sigma-Aldrich, Germany) for 1 h at 37°C. The spots were developed with a tetramethylbenzidine peroxidase substrate system (TMB, KPL, Kirkegaard & Perry Laboratories, Inc., USA).

### SDS-PAGE, Coomasie blue staining and Western blotting for IgY Abs characterization

Purified egg yolk IgY Ab pools and Wa HRV (from cell culture concentrated by ultracentrifugation and purified by cesium chloride gradient) were resuspended in Laemmli sample buffer and boiled for 10 min. The purified IgY Ab samples were subjected to sodium dodecyl sulfate-polyacrylamide gel electrophoresis (SDS-PAGE) in a 12% gel and stained with Coomasie blue (Sigma-Aldrich, Germany). The concentrated Wa HRV was also subjected to SDS-PAGE in a 12% gel and and then blotted onto a nitrocellulose membrane (Bio-Rad, USA). The membrane was blocked for 1 h with PBS-Tween (0.05%) containing 3% skim milk. To test the recognition by Wa HRV specific and VP6 specific IgY Abs, the membrane was divided in pieces and incubated with Wa HRV specific IgY pool A (1∶200 dilution), Wa HRV specific IgY pool B (1∶1000) or VP6 IgY pool (1∶1000 dilution) in PBS-Tween 20 (0.05%)–skim milk (1%) overnight at 4°C, and then the membrane was washed with PBS- Tween_20_ (0.05%) and incubated 1 h at 37°C with a peroxidase labeled goat anti-IgY Ab (Sigma-Aldrich, Germany) at a 1∶5000 dilution in PBS- Tween_20_ (0.05%)–skim milk (1%). Finally, the membrane was incubated with 3,3′-diaminobenzidine (DAB; KPL, USA) for 1 h at room temperature.

### Statistical analysis

Fisheŕs exact test was used to compare proportions of animals with diarrhea and virus shedding among groups. The Kruskall-Wallis rank sum (non-parametric) test was used to compare days of onset and duration of diarrhea and virus shedding, cumulative diarrhea scores and cumulative titers of virus shed (area under the curve, AUC) among groups that were recorded from 0 to 21 PID. Neutralizing and isotype-specific Ab titers were log_10_-transformed prior to statistical analysis. Negative samples at a dilution of 1∶4 were assigned an arbitrary Ab titer of 2 for the calculation of geometric mean titers (GMTs). Differences in Ab titers among groups were evaluated by comparison of means at four different time-points post virus inoculation. Multiple comparison test of repeated measures throughout time was done following Akaike criteria for the selection of covariance matrices [44], [45]. In further comparisons of treatments and post virus inoculation time-points, Šidák´s correction was applied [46]. At 21 PID, the ASC numbers were compared among groups using the Kruskall-Wallis rank sum test. Statistical significance was assessed at p<0.05 for all comparisons. Statistical analyses were conducted using STATISTIX 8.0 (Analytical Software, USA) and MedCalc® version 11.1.1.0 statistical software.

## Results

### Chicken egg yolk IgY Abs specifically recognized Wa HRV and showed virus neutralization activity

After the first immunization, all hens immunized with Wa HRV and VP6 recombinant protein developed Ag specific serum Abs. The mean serum IgY Ab titer to Wa HRV was 16,384 for all the hens, independent of the antigen used for the hyperimmunization at two weeks after the first immunization. Around the third week after the first dose, hens started laying eggs. The egg yolks from Wa HRV hyperimmunized hens had high IgY Ab titers to Wa HRV with an Ab titer of 16,384 in the crude egg yolk extract (1∶3 in distilled water, data not shown). On the other hand, the egg yolks from VP6 hyperimmunized hens had IgY Ab titers to Wa HRV of 4096 (data not shown).

All the animals received a second immunization after 30 days (4 weeks) and IgY Ab titers to Wa HRV in sera and egg yolks rose to 262,144 and 65,536; respectively in the Wa HRV hyperimmunized group. The serum Wa HRV specific IgY Ab titers were always higher than the Ab titers detected in crude egg yolk extracts. Hens received 7 boosters during the 44 weeks of the experiment and the average Wa HRV IgY Ab final titers were 262,144 and 4096, in serum and crude egg yolk extract, respectively. For VP6 hyperimmunized hens, the average Wa HRV IgY Ab titer in serum was 65,535 and in egg yolk extracts was 4096 (data not shown).

**Figure 1:**
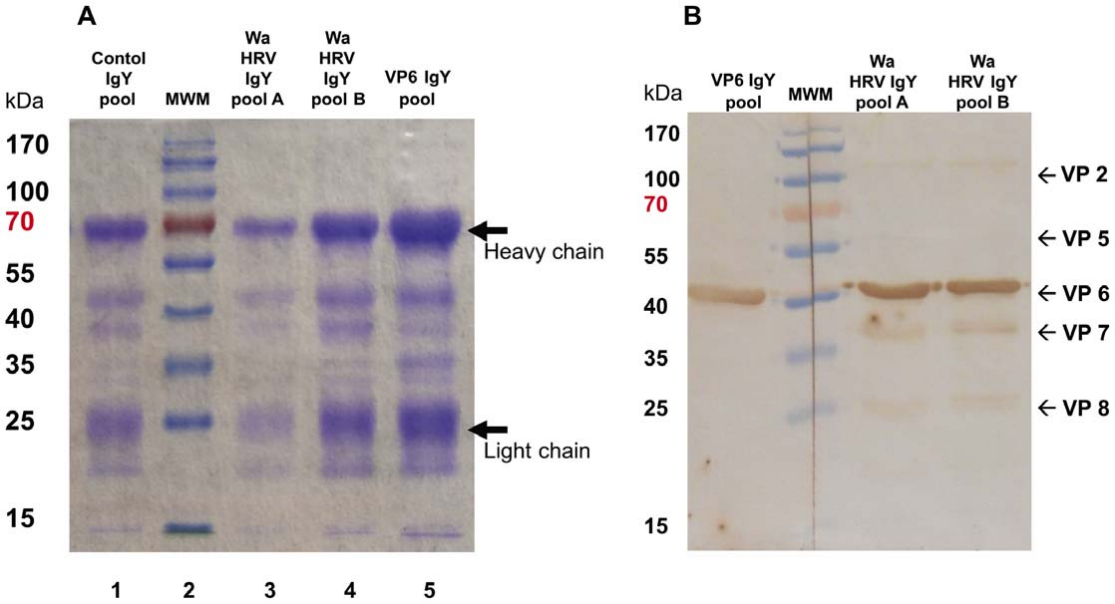
*In vitro* characterization of VP6 specific and Wa HRV specific chicken egg yolk IgY Abs and control IgY Abs used for this experiment. **A-** Coomasie blue stained SDS-PAGE of IgY Abs in control IgY (line 1), Wa HRV pool A (line 3) and pool B (line 4) and VP6 IgY pool (line 5). Total protein loaded in each lane: Lane 1 = 19.2 µg; Lane 3 = 18.8 µg; Lane 4 = 28.1 µg; Lane 5 = 31.2 µg. Lane 2: molecular weight marker (MWM; Fermentas, Thermo Fisher Scientific Inc., USA). Arrows indicate the light and heavy chain of IgY Abs. **B-** Immunoblot analysis of Wa HRV SDS-PAGE incubated with VP6 IgY pool or Wa HRV IgY pool A and B. Approximately 5 µg of purified Wa HRV were loaded in the SDS-PAGE. The SDS-polyacrylamide gel was transferred to a nitrocellulose membrane and then one segment was incubated with Wa HRV specific IgY Abs from pool A (1∶200 dilution from the stock), another segment was incubated with Wa HRV specific IgY Abs from pool B (1∶1000 dilution from the stock) and the third segment was incubated with VP6 IgY pool (1∶1000 dilution from the stock). After washing, the segments of membrane were incubated with peroxidase label goat anti chicken IgY polyclonal serum (Jackson ImmunoResearch Laboratories Inc.; USA). The Western blot assay was developed with 3,3′-diaminobenzidine (DAB).

ELISA and VN Ab titer to Wa HRV in the IgY and IgG Ab pools are detailed in Table 2. Total protein concentration and total IgY concentration in the pools were also determined. There was a direct positive correlation between the total IgY concentration and the total protein concentration of the pools. The IgY pools were analyzed by Coomasie blue staining. As shown in Figure 1A, all the pools had bands at 25 KDa and 70 KDa, corresponding to the light and heavy chains of IgY Abs, respectively. Other bands are also present in all the lanes, corresponding to other components of egg yolk that co-precipitated under the applied semi-purification protocol. They represent smaller fractions of the total protein solutions obtained (Figure 1A).

The generated IgY Abs recognized Wa HRV in immunoblot assay, as shown in Figure 1B. The IgY Abs from Wa HRV hyperimmunized hens recognized mainly VP6 (45 kDa), that represents the major viral protein, and also other viral proteins like VP2, VP7, VP5* and VP8* (Figure 1B, right panel). On the other hand, the Abs obtained from VP6 hyperimmunized hens specifically recognized VP6 protein from HRV while the other viral proteins, including neutralizing antigens, were not recognized in concordance with the low VN activity detected in this pool, that was similar to that of the control IgY (Figure 1B, left panel).

Thus, Lohmann Brown Classic laying hens developed Wa HRV specific IgY Abs in serum after hyperimmunization with this antigen or with the viral protein VP6 and these Abs were effectively transferred to the egg yolks. Furthermore, these IgY Abs to Wa HRV were semi-purified by salt-precipitation, without losing their ability to recognize Wa HRV (ELISA and VN assay and under denaturalizing conditions in Western blot). The IgY Abs from Wa HRV hyperimmunized hens recognized critical virus neutralizing antigens (VP7, VP5* and VP8*) in Western blot and demonstrated virus neutralizing activity against Wa HRV by VN assay. The IgY Abs from VP6 hyperimmunized hens also recognized Wa HRV by ELISA but failed to neutralize the Wa viral infection in VN assay (Table 2 and Figure 1).

### Egg yolk IgY Abs confer significant protection rates against Wa HRV diarrhea in a dose-dependent manner

**Figure 2:**
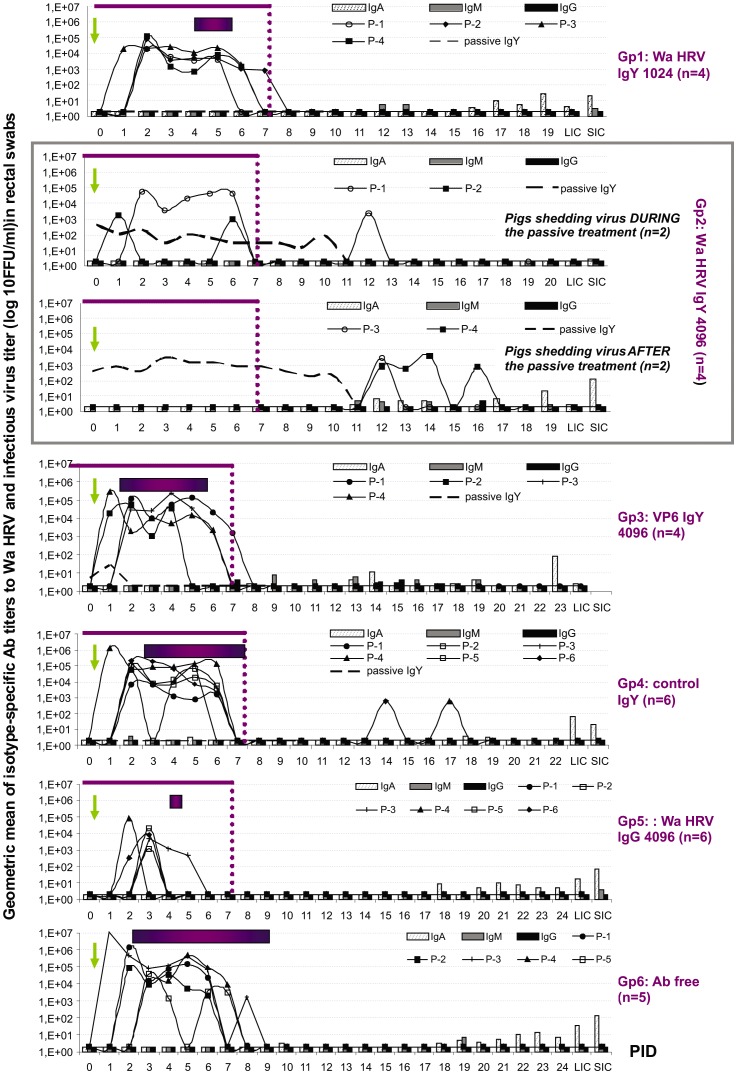
Geometric mean isotype-specific Ab titers (GMT) to Wa HRV per group and mean titer of virus shed daily per pig (from CCIF assay). SIC: small intestinal contents; LIC: large intestinal contents euthanasia. Horizontal bars represent the mean duration of diarrhea (score >2). The arrow at 0 PID indicates VirWa HRV inoculation day. Thin line: duration of the passive treatments. Dashed line: IgY Abs in rectal swabs. Each pointed line represents the virus shed by individual pigs in each group.

Results of the parameters studied to evaluate the protection against diarrhea and virus shedding are summarized in Table 3. The time course of the infection, detection of passive Ab treatment and profile of the local Ab response for each treatment group is depicted in Figure 2. All piglets in the negative control groups (Gp6: Ab free milk and Gp4: control IgY) became infected shortly after oral VirHRV Wa challenge and developed diarrhea. The severity of the illness was significantly lower in control IgY treated piglets (Gp4: 14.5) than in the Ab free milk group (Gp6: 20.2), but still significantly higher than those in the experimental groups of pigs that received RV-specific Ab treatments (Gp1: 6.8; Gp2: 7.0 and Gp5: 5.3). On the other hand, the group of piglets treated with VP6 IgY Abs (Gp3: 14.5) also developed diarrhea of a statistically similar mean severity to that observed in control IgY treated animals (Gp4).

**Table 3:** Diarrhea and virus shedding in gnotobiotic piglets after oral inoculation with VirWa HRV (P[8]G1).

Treatment Group	n	DIARRHEA+				VIRUS SHEDDING*			
		% Affected Animals	Mean Onset (PID)	Mean Duration (days)	Mean Severity#	% Affected animals	Mean Onset (PID)	Mean Duration (days)	AUC
**Gp1: Wa HRV IgY 1024 milk**	4	100%^A^	4.0^B^	1.5^A^	6.8^A^	100%	1.8^A^	5.3^BC^	9.7×10^4AB^
**Gp2: Wa HRV IgY 4096 milk**	4	0%^B^	NA^C^	0.0^A^	7.0^A^	100%	7.0^B^	3.0^AB^	4.5×10^4A^
**Gp3: VP6 IgY 4096 milk**	4	100%^A^	1.8^A^	4.0^B^	16.0^B^	100%	3.7^A^	5.8^BC^	2.7×10^5B^
**Gp4: CONTROL IgY milk**	6	100%^A^	3.3^AB^	4.5^B^	14.5^B^	100%	2.2^A^	5.0^BC^	4.7×10^5B^
**Gp5: Wa HRV IgG 4096 milk**	6	33%^AB^	4.0^B^	0.7^A^	5.3^A^	83%	3.0^A^	1.6^A^	1.9×10^4A^
**Gp6: Ab free milk**	5	100%^A^	2.4^A^	6.8^B^	20.2^C^	100%	2.2^A^	6.0^C^	2.9×10^6B^

Abbreviations: n = number of animals per group; AUC: area under the curve.

* Determined by ELISA and CCIF.

+ Diarrhea duration was defined as the number of days with fecal score ≥2. Stool consistency was scored daily (0 = normal; 1 = pasty; 2 = semi-liquid; 3 = liquid).

# Mean severity: mean cumulative scores from 0 to 21 PID (sum daily fecal score)/n. Proportions or means in the same column with different superscript upper case letters differs significantly (Fisheŕs exact test, p<0.05 and Kruskall-Wallis rank sum test; p<0.05). Piglets were fed with 210 ml of sterile milk, twice a day, for 9 days with or without the addition of the corresponding volume of Wa HRV IgY pools, VP6 IgY pool, control IgY pool or Wa HRV porcine IgG pool, according to the experimental group.

As expected for a local treatment with homologous passive maternal Abs (Gp5: Wa HRV IgG 4096, positive control group), the protection conferred by HRV-specific porcine IgG Abs at a final ELISA Ab titer of 4096 was very high, with only two animals with one and three days of mild HRV diarrhea, respectively and four animals shedding a low amount of virus asymptomatically for a few days (mean: 1.6 days).

The supplementation of milk diet with Wa HRV IgY Abs at a final ELISA Ab titer of 4096 for 9 days protected 100% of the animals (4/4) against virulent HRV-associated diarrhea (Gp2, Table 3). Piglets in this group shed virus asymptomatically. The pattern of virus shedding was quite variable, with one animal shedding virus right after virus inoculation, another pig with intermittent shedding during and after the treatment; and two animals shedding only after the end of the passive treatment (Figure 2). The mean duration of virus shedding was significantly shorter compared with the negative control group (Gp2: 3.0 days and Gp6: 6.0 days, respectively).The magnitude of the infection in Gp2 (considered as the amount of virus shed determined as the area under the shedding curve (AUC: 4.5×10^4^) was comparable with the AUC in the Wa HRV IgG 4096 treated piglets (Gp5: 1.9×10^4^) and significantly less than that in piglets from negative control groups (Gp6 Ab free milk: 2.9×10^6^; Gp4 control IgY: 4.7×10^5^ and Gp3 VP6 IgY: 2.7×10^5^). Finally, pigs receiving Wa HRV IgY at a final IgY Ab titer of 1024 (Gp1) became infected shortly after virus inoculation but developed significantly less severe HRV-associated diarrhea of shorter duration (Gp1: 1.5 days), compared with the Ab free negative control group (Gp6: 6.8 days) demonstrating that the protection was dose-dependent (Table 3).

Collectively the passive supplementation of the milk diet with Wa HRV IgY Abs at a final Ab titer of 4096 for 9 days resulted not only in the prevention of clinical symptoms, but also in a reduction of the infectious virus shed to the environment (delayed onset, shorter duration and lower AUC of virus shedding).

### The profile of the serum and local Ab responses to Wa HRV infection was not affected by the oral administration of passive Abs

**Figure 3:**
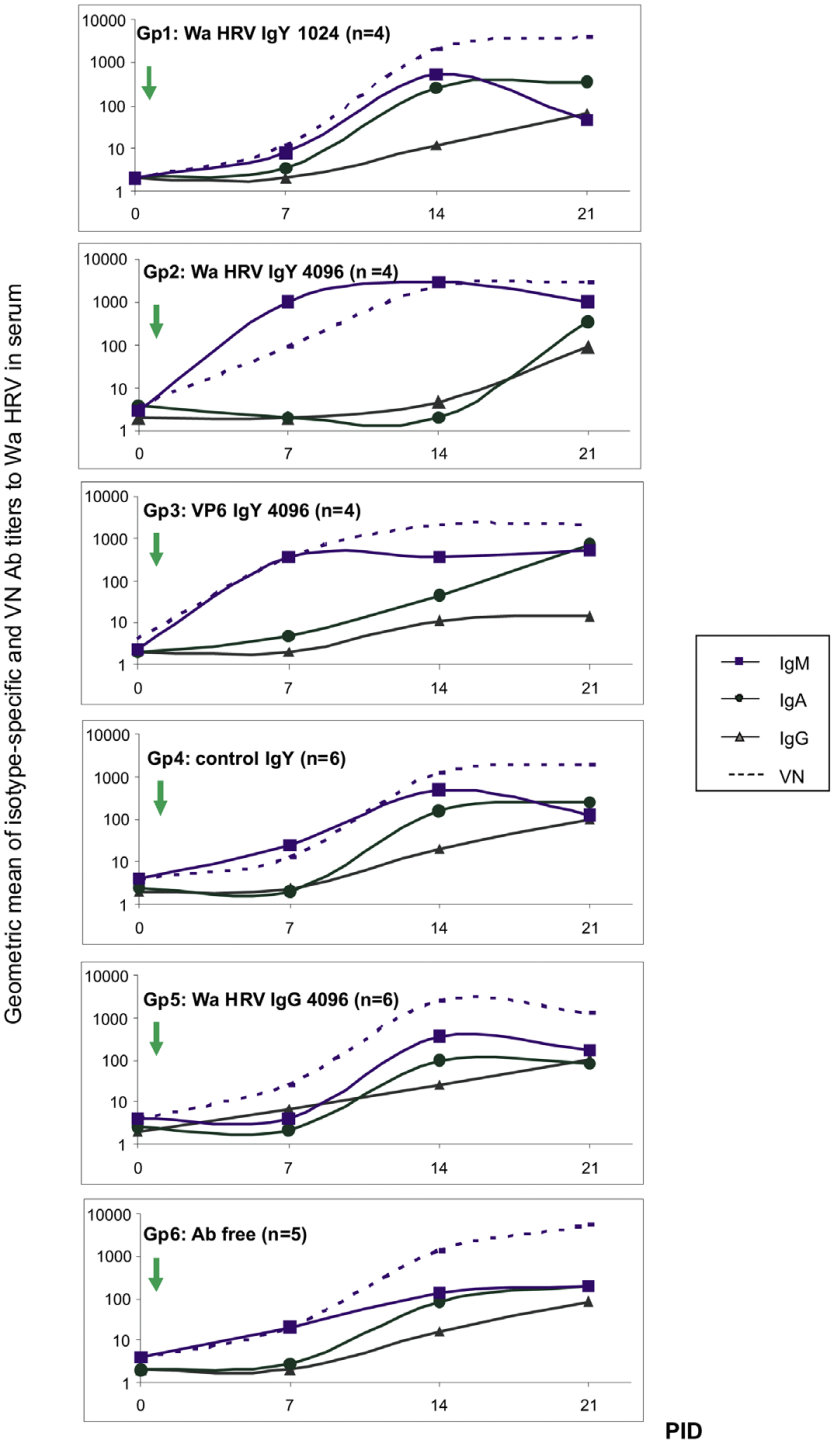
Wa HRV specific porcine Abs in piglet´s sera during the experience. The isotype-specific and VN Abs to Wa HRV in serum samples were determined weekly by ELISA and VN assay. The arrow indicates the experimental inoculation with VirWa HRV (0 PID). PID: post inoculation days.

A critical goal for a passive treatment to HRV is to prevent the illness without interfering with the development of the active immune response. As expected for colostrum deprived animals, all the pigs tested negative for Abs at 0 PID (72 h of age). This was a critical point for the experimental design, making piglets an ideal model to study the modulation caused by a passive local Ab treatment on the antigen-induced immunity *in vivo*. According to a primary immune response to RV infection, the first isotype detected in serum was IgM from 7 PID on in all groups (Figure 3). The presence of IgM Abs was associated with VN activity, as can be seen at 7 PID where this was the only isotype detected in serum (Figure 3). The IgM Ab response peaked at 14 PID in all groups and Gp2 (Wa HRV IgY 4096) had significantly higher titers than all the other groups of piglets. A similar profile can be seen for VN Abs at this experimental time (Figure 3).

The IgA Ab response to Wa HRV in serum samples started at 14 PID and was closely followed by IgG Abs, except for piglets in Gp2 (Wa HRV IgY 4096) that did not have IgA Abs at 14 PID but still had IgM Abs at this experimental time point, a profile that remained until the end of the experiment (Figure 3). Finally, the higher titers of IgG Abs were detected at 21 PID, together with IgA and IgM Abs, and were associated with high VN activity (Figure 3).

We also explored how the passive treatments affected the development of the local (gut) isotype specific Ab responses to Wa HRV after Vir Wa challenge (Figure 2). The main isotype detected in the rectal swabs and intestinal contents was IgA reaching the peak Ab titer at 21 PID. No significant differences were observed in the IgA Ab titers among any of the experimental groups during the experiment (Figure 2). A similar situation was observed for IgM and IgG porcine Abs to Wa HRV, where the highest titers were detected in rectal swab fluids at 21 PID, but not significantly so (Figure 2).

The presence of IgY Abs to Wa HRV was also determined by ELISA. No Wa HRV-specific IgY Abs were detected in the serum samples from any of the IgY treated piglets from Gp1, Gp2 and Gp3 (data not shown). However, these heterologous Abs were detected in rectal swabs from pigs in Gp2 (Wa HRV IgY 4096 milk) and Gp3 (VP6 IgY 4096 milk), while none of the samples from piglets in Gp1 (Wa HRV IgY 1024 milk) tested positive. Interestingly, in Gp2 pigs, Wa HRV IgY Abs were detected during the treatment and until 3 to 4 days after the cessation of the passive treatment (around 11 PID) (Figure 2). However, no IgG Abs were detected in the rectal swabs from Gp4 (Wa HRV IgG 4096) during the time of the passive supplementation (Figure 2).

### The oral administration of IgY Abs induced a porcine IgG Ab response in the serum and local specific IgA and IgG Abs in neonatal piglets

**Figure 4:**
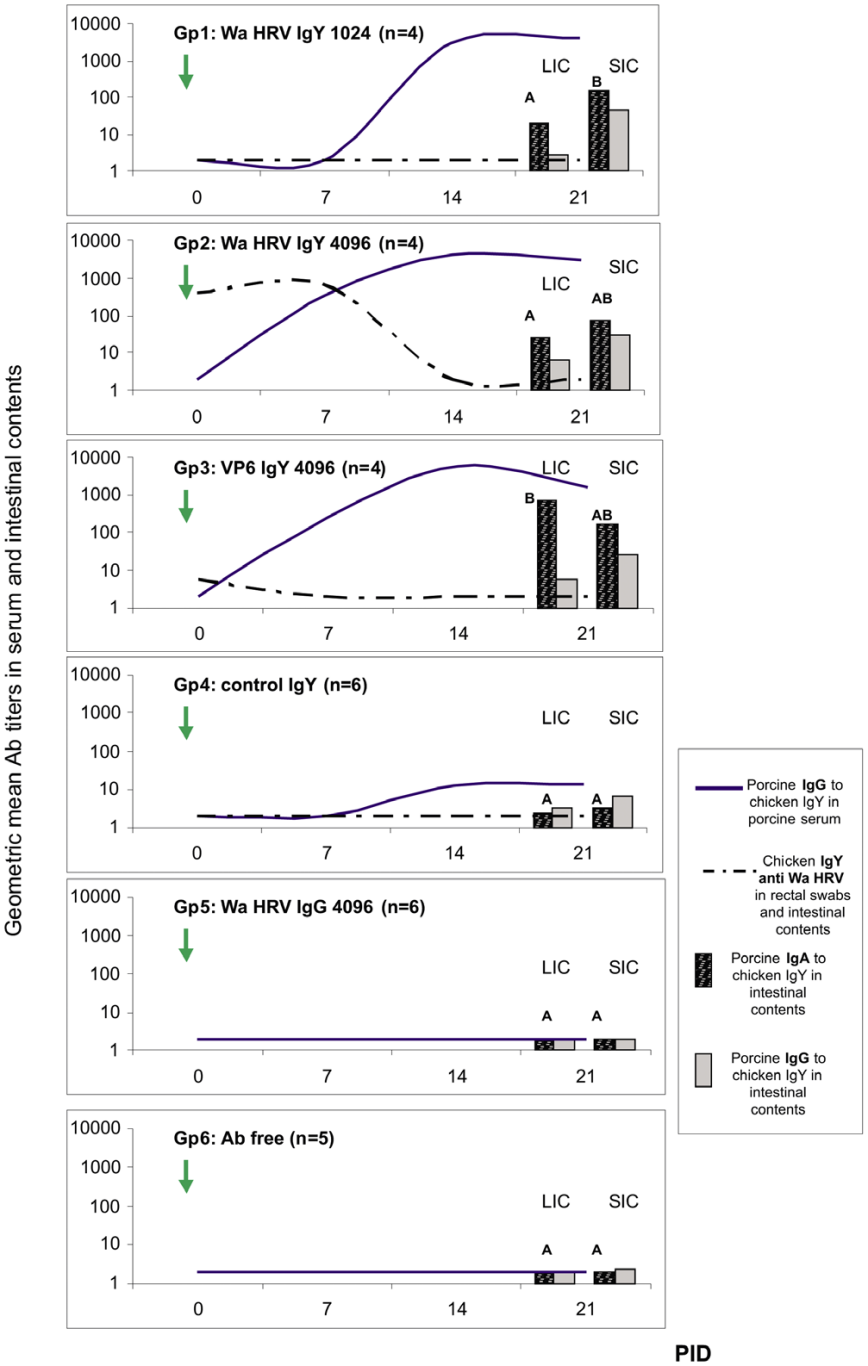
Porcine immune responses to chicken IgY passive treatments. Lines represent geometric mean of porcine IgG Ab titers (GMT) to chicken IgY in serum samples and passive IgY to Wa HRV Abs in rectal swabs per group every 7 days. The bars at 21 PID represent porcine IgG and IgA Abs to chicken IgY in the intestinal contents, where SIC: small intestinal contents and LIC: large intestinal contents, that were collected after euthanasia. All animals were orally inoculated at 72 h of age [0 post inoculation day (0 PID)], and euthanized at 21±3 PID. The arrow at 0 PID indicates VirWa HRV inoculation day. Different letters indicate significant differences between groups (p<0.05).

Although no Wa HRV-specific IgY Abs were detected in the serum samples from any of the IgY treated piglets (Gp1, Gp2 and Gp3), all the animals that received milk supplemented with IgY Abs developed IgG Abs to chicken IgY in serum (Figure 4). As shown in Figure 4, piglets in Gp2 and Gp3 (Wa HRV IgY 4096 milk and VP6 IgY 4096 milk, respectively) developed IgG Abs to IgY at 7 PID (9 days after starting the passive treatment). At 14 PID, all the IgY treated piglets had specific IgG Abs in serum, with statistically higher titers in Gp3 (VP6 IgY 4096 group). Finally, at 21 PID all the piglets in Gp1, 2 and 3 receiving heterologous Ab treatments showed similar IgG Ab titers to IgY in serum that were significantly higher than the IgG Ab titer to IgY in Gp4 piglets (control IgY milk). As expected for the piglets that did not received heterologous immunoglobulins, no IgG Abs to IgY were detected in Gp5 and Gp6 piglets (Wa HRV IgG 4096 milk and Ab free milk, respectively) (Figure 4).

At 21 PID, intestinal contents from all the pigs were obtained and IgA and IgG Abs to IgY were also determined by ELISA. A similar profile and magnitude of the immune response were observed in the intestinal tract. As shown in Figure 4, IgA Abs to IgY were the most abundant isotype present in small and large intestinal contents. All the piglets in Gp1, 2 and 3 showed local responses to the passive treatment, and VP6 IgY treated animals had statistically higher titers of IgA Abs to IgY in LIC than pigs in the other groups. Once again, piglets in Gp4 (control IgY milk) had almost no detectable Abs to IgY in these samples. Pigs in Gp5 and Gp6 (Wa HRV IgG 4096 milk and Ab free milk, respectively) were negative for Abs to IgY (Figure 4).

### The oral administration of semi-purified passive heterologous IgY Abs did not affect the isotype profile of the Ab secreting cell responses to Vir Wa HRV infection but was associated with significantly fewer numbers of HRV–specific IgA ASC in the duodenum

**Figure 5:**
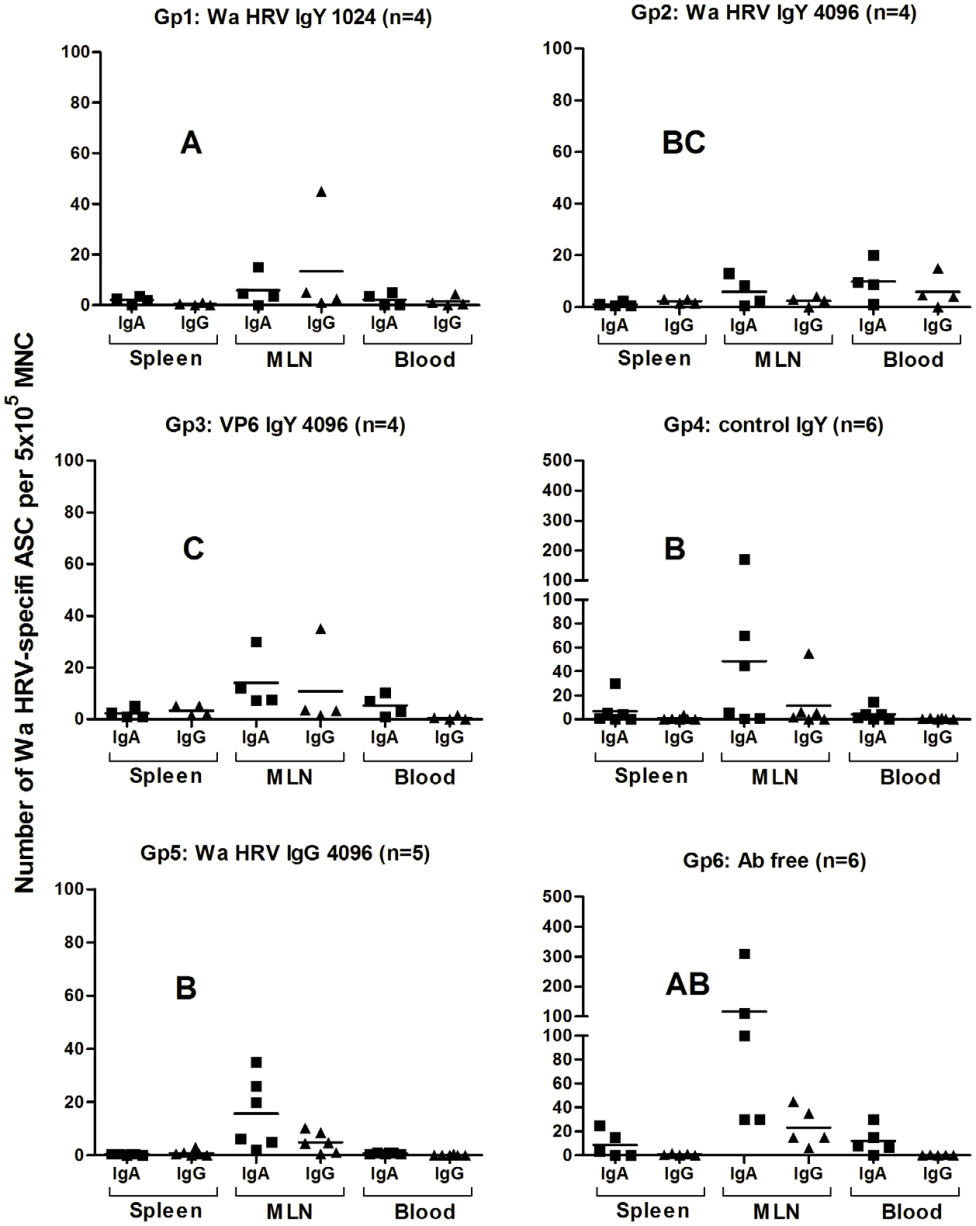
Numbers of Wa HRV-specific ASC per 5×10^5^ MNC obtained from systemic lymphoid tissues (Blood and Spleen) and MLN draining the small and large intestine at 21 PID. For each tissue, when comparing ASC numbers of the same isotype among treatment groups different letter indicate a significant difference (Kruskal-Wallis rank sum test, p<0.05). n = number of piglets in each group. The IgM ASC response was not included as no IgM ASC were detected in most of the groups of pigs.

**Figure 6:**
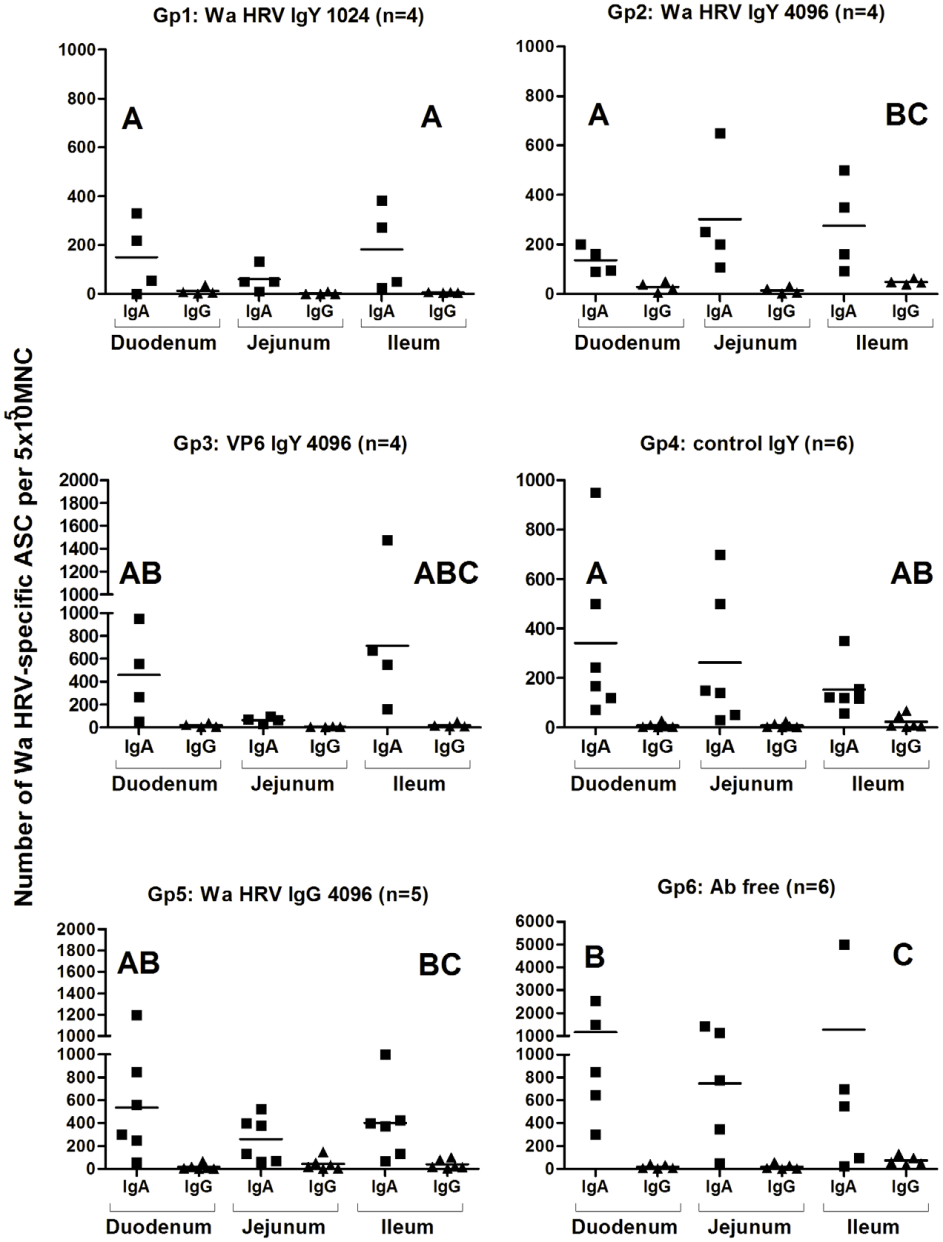
Numbers of Wa HRV-specific ASC per 5×10^5^ MNC obtained from gut-associated lymphoid tissues (Duodenum, Jejunum, Ileum) at 21 PID. For each tissue, when comparing ASC numbers of the same isotype among treatment groups different letter indicate a significant difference (Kruskal-Wallis rank sum test, p<0.05). n = number of piglets in each group. The IgM ASC response was not included as no IgM ASC were detected in most of the groups of pigs.

Antibody secreting cell (ASC) responses to Wa HRV at 21 PID were mainly distributed along the different portions of the intestinal lamina propria (duodenum, jejunum and ileum), followed by the mesenteric lymph nodes. Fewer ASC were detected in systemic lymphoid tissues (blood and spleen) for all the experimental groups of pigs. The main isotype detected was IgA followed by IgG. Low numbers or no IgM ASC were detected in all the tissues from the piglets and so the numbers are not shown (Figures 5 and 6).

In the systemic tissues (blood and spleen) and the MLN, the IgA and IgG ASC counts were mostly comparable among all the experimental groups of pigs. Only IgG ASC from blood showed statistical differences whereby piglets from Gp3 (VP6 IgY 4096) exhibited the highest counts (Figure 5).

The IgA ASC responses to HRV in the MLN of the piglets receiving HRV- and VP6-specific IgY (Gp1, 2 and 3, respectively) were lower than the responses detected in the other groups of piglets, but not significantly so (Figure 5).

Regarding the GALT, in the duodenum, Wa HRV specific IgA ASC counts in three of the groups of piglets receiving IgY Ab treatments (Gp1, Gp2 and Gp4) were also statistically lower than those detected in pigs fed Ab free milk (Gp6). Piglets in Gp3 (VP6 IgY 4096 milk) showed an intermediate count of IgA ASC in duodenum and it was similar to that observed in Gp5 pigs (Wa HRV IgG 4096 milk). Although no statistically significant differences were recorded, the IgA ASC counts in jejunum were especially high in Gp6 (Ab free milk) compared with the other groups of pigs (Figure 6).

A similar trend, but of less magnitude, was observed for Wa HRV-specific IgG ASC in the ileum (Figure 6). The group of piglets fed milk without Abs showed an ASC response of higher magnitude than any of the other experimental groups of animals (Figure 6).

## Discussion

Passive treatments against gastrointestinal pathogens such as RVA need to be safe, economical and highly specific. It has been reported that IgY Abs from hens are an excellent source of polyclonal Abs against RVA and other pathogens [29]. However, most of these experiments failed to determine the protective dose and their effect on the development of the immune response to RVA or to the IgY itself [10], [25], [47], [48]. In the present work, we focused not only on the generation and characterization of Wa HRV IgY Abs, but also on their ability to confer protection against VirWa HRV infection when administered only as a milk supplement at different Ab titers in a gnotobiotic pig model, the only animal model susceptible to human RVA infection and disease. Furthermore, we followed the animals to characterize the possible interference of the administration of semi-purified egg yolk Abs with the neonatal humoral immune responses to HRV after experimental VirWa challenge and the host immune responses to the passive treatment administered.

As shown, Wa HRV specific IgY Abs were obtained from hyperimmunized hens. The difference between the Wa HRV Ab titer in hens' sera (262,144) and egg yolks (65,536) can be due to a saturation of the active transport of IgY from serum to the egg yolk, which depends on the expression and activity of FcRY receptor. The IgY Abs obtained were characterized by ELISA, VN, Coomasie blue staining and immunoblot assay. The polyclonal Abs generated recognized Wa HRV and showed VN activity. The supplementation of the milk diet with semi-purified Wa HRV-specific IgY Abs at a final ELISA titer of 4096 (VN: 256) for 9 days protected 100% of the animals against VirWa HRV-associated diarrhea and significantly reduced virus shedding. As the treatment with a final titer of IgY Abs of 1024 failed to protect animals, it is clear that the IgY Abs protection behaves in a dose-dependent manner, as reported for other heterologous and homologous Ab sources tested in mice, calves and children [10], [24], [25], [26]. The passive treatment with IgY at a final Ab titer of 4096 in milk also modified the time course of the infection, delaying the onset of virus shedding until the end of the treatment in some of the animals and significantly reducing the duration of virus shedding. The efficacy of the treatment was associated with the detection of virus specific IgY Abs in rectal swabs of Gp2 treated animals (Wa HRV IgY 4096 milk), indicating that semi-purified IgY Abs were able to resist the environment of the neonatal gastrointestinal tract and remained biologically active. The IgY Abs in rectal swabs of Gp2 pigs showed a similar trend for all the piglets, where a decrease in HRV IgY Ab titer was associated with virus shedding. The binding of Wa HRV-specific IgY Abs with the infectious virions may explain the absence of diarrhea after this treatment, although the virus replicated in the gut of the newborn piglets. As previously reported in the literature, similar results were observed in neonatal calves fed milk supplemented with crude egg yolk at the same final IgY Ab titer determined by ELISA [26]. The results of this latter study also suggest that the administration of milk supplemented with IgY Abs at a final ELISA IgY Ab titer of 4096 (VN: 256) for a longer period of time (at least 15 days) might improve protection against virus shedding.

The passive treatment of newborn piglets with VP6 specific IgY Abs at a final Ab titer of 4096 (specifically to Wa HRV determined by ELISA, but low VN Ab titer: 64) also failed to protect animals after virus inoculation. It has been established previously that conventional IgG Abs directed to this viral protein are not neutralizing *in vitro* or *in vivo* and do not induce protection when administered as a passive oral treatment *in vivo* [49], [50], [51], [52], [53], [54], [55]. In fact, pigs in this group developed diarrhea shortly after experimental VirWa challenge and had a mean severity similar to that of the control IgY treated animals.

On the other hand, piglets fed milk supplemented with VP6 IgY and control IgY developed a diarrhea of lower magnitude/severity than that observed in Ab free milk fed piglets. This could be related to the ability of other substances in eggs from hens to prevent diarrhea as has been reported in a castor oil mouse model of diarrhea [56]. Besides the IgY, other factors that co-precipitate with the IgY may be responsible for the reduction observed in diarrhea severity. Further investigation is needed to elucidate the basis for this interesting effect.

In regard to the immunogenicity of the heterologous immunoglobulins, piglets receiving IgY treatments developed both systemic IgG and local IgA and IgG Ab responses to avian IgY. Considering the total protein and total IgY concentrations in each IgY pool (detailed in Table 2), there was a trend toward the development of higher immune responses to IgY Abs in the piglets receiving higher amounts of IgY: VP6 IgY = Wa IgY 4096>Wa IgY 1024 = control IgY. This is especially clear in the cases of Gp2 and Gp3 (Wa HRV IgY 4096 and VP6 IgY 4096 treatments, respectively). In these groups, the treatments had higher total IgY and protein concentrations and the piglets IgG Ab responses to chicken IgY in serum were statistically higher than, for instance, the control IgY treated piglets (Gp4) (Table 2 and Figure 4). Porcine responses to IgY passive treatment also behave in a dose dependent manner in regard to the total IgY concentration administered. These results are not surprising, since IgY have been reported to be highly immunogenic and it has been used as a model antigen for testing mucosal adjuvants [36], [57]. However, pigs did not show other signs associated with allergy like elevated body temperature, coughing, itching, or skin rashes (data not shown). Our results are in concordance with those reported by Torché *et al.* after local administration of IgY in pigs. They found that IgG Abs are the main subclass in serum responses whatever the administration site [57]. On the other hand, allergenicity of the IgY treatment (IgE production) was not assessed in this study and further experiments will be needed to evaluate this issue. Piglets are an excellent model for allergy and investigations performed in this animal model demonstrate hypersensitivity reactions that are more closely aligned with human responses than small animal models [58]. However it was previously demonstrated by Akita *et al.* that purified IgY and its components are highly immunogenic, but induce low IgE Ab responses in a mouse model [36].

Regarding the B-cells immune response to Wa HRV, we studied not only the isotype of specific Abs responses in serum and rectal swab fluids, but also the distribution, isotype and profile of Wa HRV specific ASC in systemic and GALT. Previous studies have demonstrated that the administration of passive treatments based on homotypic or heterotypic Abs can modulate the immune response to RV [18], [19], [24], [26]. In the present work, the administration of IgY Abs did not appear to interfere with the development of the immune response to Wa HRV, as Abs in serum and rectal swabs showed comparable profiles for all the experimental groups of pigs. At 21 PID, the ASC response was mainly detected in GALT, as expected. The main isotype of Wa HRV-specific ASC detected at 21 PID was IgA in all the tissues, in agreement with the presence of HRV-specific IgA Abs in feces and intestinal contents at this experimental time point. The detection of higher numbers of ASC in the gut of Gp6 piglets (Ab free milk) could be associated with higher VirHRV Wa replication in the unprotected piglets. Our results indicate that pigs receiving passive Ab treatments had the same isotype profile of IgA ASC but of lower magnitude than piglets fed Ab free milk. However, the response observed was strong enough to prevent Wa HRV diarrhea after a second exposure to the virus, as has been previously reported [20]. In the groups showing high protection rates against diarrhea and/or virus shedding (Gp2 and Gp4) this effect could be associated with less severe damage to the intestinal mucosa. However in pigs receiving Wa HRV IgY 1024 milk, VP6 IgY 4096 milk and control IgY milk (Gp1, 3 and 4, respectively) the presence of lower numbers of HRV specific ASC are difficult to explain, as they all developed diarrhea after VirHRV Wa inoculation.

Collectively, these results demonstrate that a passive treatment based on IgY Abs at a final Ab titer of 4096 not only prevents diarrhea but also significantly reduces the amount of infectious virus shed (compared with negative control groups), without interfering with the development of the immune response against VirWa HRV.

Previous studies by our group indicate that the passive treatment of newborn calves for 14 days with crude egg yolk containing IgY Abs against bovine RV at a final Ab titer of 4096 also induced high rates of protection against Virulent bovine RV challenge. In addition, a positive modulation of the GALT associated ASC immune response to RV was observed in all the animals that received egg yolk as a supplement in the milk diet, independently of the specificity of the IgY Abs [26]. These findings may have implications for the use of crude egg yolk or spray dried egg powder as a source of heterologous Abs. In the present work IgY Abs were semi-purified, since the evaluated treatment was designed to be administered to human infants in the future. The results showed that semi-purified IgY Abs (IgY control) may have less enhancing effects on the local immune response to HRV. Low molecular weight co-factors may have co-precipitated during the IgG and IgY Ab semi-purification. Thus other components of the egg yolk may be responsible for the immune modulation previously reported [26], [59], [60].

There is a clear need to define improved, cost-effective interventions in the management of diarrhea due to RV until the time an effective, safe, and inexpensive vaccine, affordable to developing countries, is available [25]. Unfortunately, diarrhea treatment is not a priority in many countries. Diarrhea mortality rates dropped by 75% from 1980 to 2008, but remain unacceptably high [1]. New interventions for treatment and RV vaccines for prevention of diarrhea have provided an opportunity to revitalize diarrhea-control programs worldwide [2]. To our knowledge, this is the first time that a protective dose of IgY Abs administered orally as a milk supplement has been assessed in an animal model that more closely mimics the physiology of human infants. This strategy can be easily scaled-up and egg yolk represents an inexpensive source of large amounts of polyclonal IgY Abs [25]. Further studies are needed to formulate maternal milk for pediatric use and to determine the safety, stability and administration regime of this passive treatment based on IgY Technology. Additional studies in animal models are necessary to evaluate allergic responses to the treatment in different doses, including IgE Ab responses. The administration of IgY Abs should not be recommended to known egg-allergic patients. However, because of the high morbidity and mortality of HRV associated diarrhea in developing countries and among immunesuppressed patients, the potential risk of sensitizing these patients to egg during infancy or an episode of frequently fatal gastroenteritis must be balanced against their potential benefit as determined by appropriate clinical trials [61]. Alternatively, the administration of chicken IgY Abs to breast fed children may be of less concern, since important immune molecules such as TGF-β present in human maternal milk has been shown to be a strong anti-allergenic factor inducing oral tolerance to dietary antigens in neonates [62]. Finally, IgY Abs have been used in randomized, placebo-controlled clinical trials carried out by Sarker *et al.* and by Rahman *et al.* in hospitalized children. Sarker *et al.* reported that the treatment with semipurified IgY Abs against four HRV strains resulted in a modest improvement of diarrhea associated with earlier clearance of HRV from stools [25]. Rahman and collaborators observed earlier termination of intravenous fluid therapy by 3 days, earlier recovery from diarrhea by 2 days and earlier cessation of RV shedding in stool by 1 day [63]. In summary our results, together with the outcome of the cited clinical trials, clearly indicate the potential value of IgY Abs for the treatment of acute RV infection in pediatric patients.
